# Recovery of Homogeneous Platinoid Catalysts from Pharmaceutical Media: Review on the Existing Treatments and the Perspectives of Membrane Processes

**DOI:** 10.3390/membranes13080738

**Published:** 2023-08-17

**Authors:** Adrien Magne, Emilie Carretier, Lilivet Ubiera Ruiz, Thomas Clair, Morgane Le Hir, Philippe Moulin

**Affiliations:** 1 Aix Marseille Univ., CNRS, Centrale Marseille, M2P2 UMR 7340, Equipe Procédés Membranaires (EPM), Europole de l’Arbois, BP80, Pavillon Laennec, Hall C, 13545 Aix en Provence Cedex, France; adrien-amu.magne@sanofi.com (A.M.); emilie.carretier@univ-amu.fr (E.C.); 2 Sanofi Chimie, Laboratoire Génie des Procédés 1, Process Engineering, Global Chemistry Manufacturing & Control (CMC), 45 Chemin de Mételine, 04200 Sisteron, France; lilivetaracelis.ubieraruiz@sanofi.com (L.U.R.); thomas.clair@sanofi.com (T.C.); morgane.lehir@sanofi.com (M.L.H.)

**Keywords:** platinoid, homogenous catalyst, metal recovery, organic solvent, membranes

## Abstract

Catalyst recovery is a major challenge for reaching the objectives of green chemistry for industry. Indeed, catalysts enable quick and selective syntheses with high reaction yields. This is especially the case for homogeneous platinoid catalysts which are almost indispensable for cross-coupling reactions often used by the pharmaceutical industry. However, they are based on scarce, expensive, and toxic resources. In addition, they are quite sensitive and degrade over time at the end of the reaction. Once degraded, their regeneration is complex and hazardous to implement. Working on their recovery could lead to highly effective catalytic chemistries while limiting the environmental and economic impacts of their one-time uses. This review aims to describe and compare conventional processes for metal removal while discussing their advantages and drawbacks considering the objective of homogeneous catalyst recovery. Most of them lead to difficulty recycling active catalysts due to their ability to only treat metal ions or to chelate catalysts without the possibility to reverse the mechanism. However, membrane processes seem to offer some perspectives with limiting degradations. While membranes are not systematically the best option for recycling homogeneous catalysts, current development might help improve the separation between pharmaceutical active ingredients and catalysts and enable their recycling.

## 1. Introduction

Platinoids are almost indispensable in the pharmaceutical industry for synthesizing new molecules while respecting the objectives of green chemistry. It is important to be aware of the benefits, the uses, and the problems linked to these catalysts in order to understand the challenges of their recovery and reuse. This aim is even more pertinent given the scarcity and high buying costs of platinoids on one hand and the complexity of regeneration processes for these catalysts on the other.

### 1.1. General Information about Platinoids

Platinoids or PGMs—platinum-group metals—are composed of six noble transition metals: ruthenium (Ru), rhodium (Rh), palladium (Pd), osmium (Os), iridium (Ir), and platinum (Pt). They are regrouped for their similar properties and uses.

#### 1.1.1. Use of Platinoids in Industries

**Figure 1:**
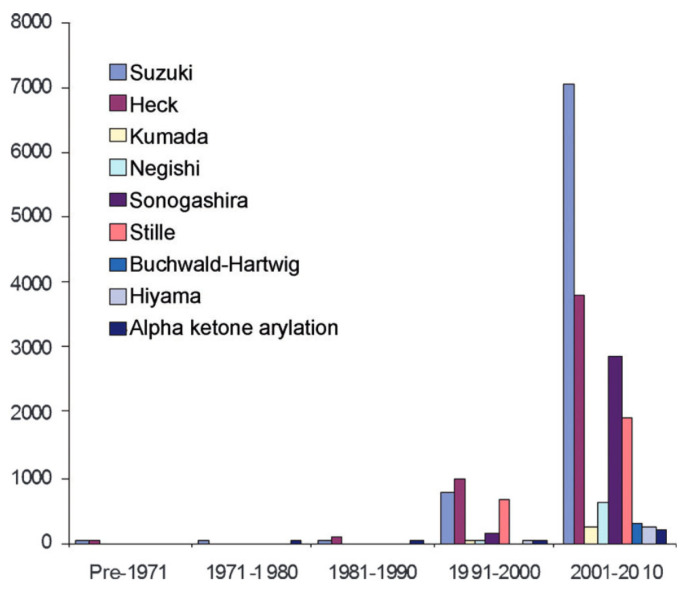
Evolution of the number of publications and patents for each decade [5].

These metals are of great interest in the industrial field and are gathered for their shared physical characteristics: corrosion and oxidation resistance, high melting points, and good catalytic capacities. Therefore, they are commonly used in different types of industries, for example, as catalytic converters in the automobile field for treating gas emissions [1,2]. In the pharmaceutical field, PGMs are used as metal catalysts [3,4]. Thanks to their high efficiency, they enable optimized chemistry that quickly goes to the targeted molecule while limiting the number of by-products and therefore the number of purification steps downstream. This meets the objectives of green chemistry. Moreover, these catalysts are almost indispensable for certain types of reactions, such as cross couplings. Cross couplings are a powerful tool for chemists as they enable the formation of new carbon–carbon bonds (C–C bonds). This set of reactions is quite recent. As shown in Figure 1, the use of cross couplings sharply increased in the beginning of the 21st century.

This led to a recognition for Richard Heck, Ei-ichi Negishi, and Akira Suzuki who won the Nobel Prize for Chemistry in 2010 [6] for these revolutionary reactions in the development of new synthesized molecules.

#### 1.1.2. Ecotoxicity and Human Toxicity

Platinoids’ properties make them toxic, especially in the environment where Pd exposure to a plant can cause growth problems by inhibiting photosynthesis and root activity [7]. With the pollution of ecosystems, the whole chain of life can be disrupted. For humans, the generated toxicity is low in the short term but above a certain concentration, allergies, hypersensitivity, and respiratory problems can emerge [8]. The toxicity of PGMs as salts may become much more important. Palladium chloride is classified as corrosive and harmful to the environment and may damage the liver, kidneys, and marrow [9]. In addition, platinoids can be bioaccumulated. This phenomenon has been proven by several studies among living organisms including humans [10,11]. This increase in concentration in living beings can be a source of greater risk in the long term. Palladium generates the greatest bioaccumulation due to its ability to easily spread in aqueous media as Pd^2+^ ions [12]. It was also discovered that the concentration of PGMs in different environments—air, water, soil, and living things—was currently increasing over the years mostly due to car emissions which pollute air and then spread to all media [12].

#### 1.1.3. Rarity and Buying Costs

Platinoids are particularly rare and palladium, for example, is present at 0.0006 ppm in the Earth’s crust [13], half as much as gold. The ore reserves are also very localized: 40% of the world’s palladium was produced by Russia in 2022 [14] and the rest by South Africa and north America. This scarcity leads to high material costs. In 2022, the price of palladium fluctuated between 50.85 and 96.98 k€ kg^−1^ [15]. However, it is one of the most affordable PGM, with rhodium prices being 5.4 times more expensive [16]. The geopolitical context can have a great influence on PGMs supplies and prices, with rising prices in recent years due to the COVID-19 pandemic and the war in Ukraine [17]. As catalysts, the buying costs of platinoids can be much more expensive. Table 1 presents three commonly used catalysts and their sales price [18].

Moreover, platinoids used in industries cause some additional costs. Indeed, their toxicity and scarcity imply a need for isolating and treating them. In the pharmaceutical industry, homogeneous catalysts such as those presented in Table 1 tend to degrade after the reaction and usually need to be replaced by fresh catalysts while sending the old ones for a complex regeneration process.

### 1.2. Platinoid-Catalyzed Cross-Coupling Reactions

Platinoid catalysts have many uses in the pharmaceutical field. In addition to their properties for enhancing yield, limiting by-products, and accelerating kinetics, platinoid catalysts are indispensable for many cyclic reactions. Olefin hydrogenations can be performed with rhodium or ruthenium catalysts such as tris(triphenylphosphine) rhodium(I) chloride [19]. Ruthenium complexes such as Grubbs catalysts are used in olefin metathesis, enabling the redistribution of chemical bonds between two alkenes [20]. But one of the most useful reaction mechanisms in the chemical industry are cross couplings which lead to C–C bonding of two reagents.

#### 1.2.1. Carbon–Carbon Bonding

**Figure 2:**
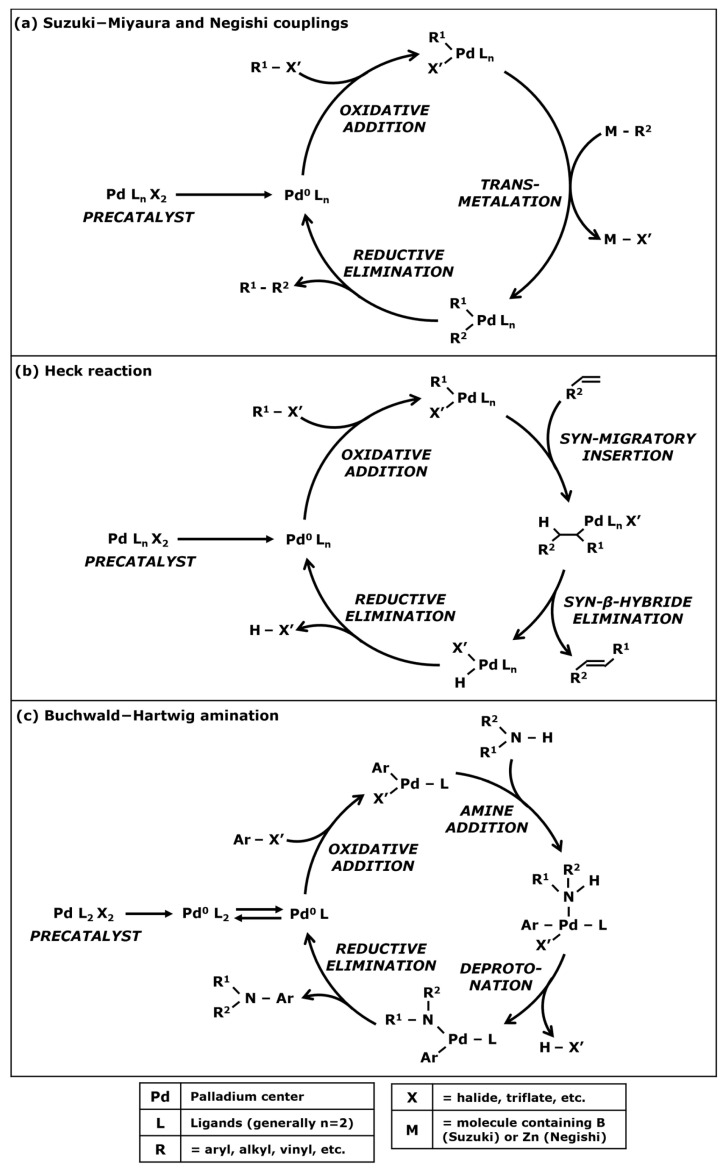
General catalytic cycle for Suzuki-Miyaura and Negishi couplings (**a**), Heck reactions (**b**), and Buchwald–Hartwig aminations (**c**).

Suzuki-Miyaura cross coupling is a well-known catalytic reaction used for generating a C–C bond between two carbonated groups—such as aryl, alkyl, and allyl—in order to create a new molecule of interest. This reaction was first published in 1979 by the Japanese scientists N. Miyaura et al. [21]. The Negishi coupling is a similar cyclic reaction also used for C–C bonding and was first published in 1976 by S. Baba and E. Negishi [22]. These two reactions were rewarded the 2010 Nobel Prize in Chemistry and are still commonly used by chemists [23,24]. Both reactions can be explained in Figure 2. The difference between Suzuki and Negishi cross couplings resides in the nature of the reagents.

These chemical cycles mostly use palladium-based catalysts. At first, the catalyst precursor—also known as the precatalyst—must be activated for having a palladium center with a zero-oxidation state. The first carbonated reagent is a halide that is fixed on the palladium center through an oxidative addition. Chlorides are often used but Suzuki and Negishi reactions can be performed with bromides or iodides [25] or even triflates [26]. The second carbonated group bonds with the palladium center through a transmetalation reaction. For a Suzuki-Miyaura cycle, this reagent is a boronic acid or a boronate and the intervention of a base is needed for performing this step. For the Negishi coupling, the carbonated group is generally an organozinc complex and does not necessitate a base. After the transmetalation step, the two carbonated groups are attached to the palladium center. A reductive elimination enables the C–C bonding between these two groups and therefore the formation of the target molecule. The catalyst returns to its zero-oxidation state and a new chemical cycle can start until the reagents are consumed.

Several other similar cyclic reactions exist with the same three steps such as the Stille reaction [27,28] using an organotin complex during the transmetalation step. Another alternative is the Sonogashira coupling [29,30] used for forming a C–C bond between a terminal alkyne and a halide. The transmetalation step involves a second catalytic cycle with a copper or a palladium co-catalyst.

The Heck reaction is the third cross-coupling that was co-awarded the 2010 Nobel Prize. R.F. Heck and J.P. Nolley first published [31] a catalyzed cross-coupling between a halide and a terminal alkene in 1972. The chemical mechanism presented in Figure 2b starts similarly with an oxidative addition of the halide on the metal center of the catalyst. However, instead of a transmetalation, the terminal alkene bonds with the metal center through a syn-migratory insertion. Then, a syn-β-hybride elimination enables the C–C bonding and the formation of the synthesized molecule. The catalytic complex goes through a reductive elimination induced by the presence of a base, enabling the return of the catalyst to its zero-oxidation state.

#### 1.2.2. Carbon–Nitrogen Bonding

In addition to enabling C–C bonding, cross couplings can be adapted for generating a bond between a carbon and a nitrogen (C–N bond) in the presence of a rare-metal catalyst. This kind of reaction could also be quite interesting in the pharmaceutical industry for the synthesis of active pharmaceutical ingredients (APIs). The Buchwald–Hartwig amination resulting in works from J.F. Hartwig and S.L. Buchwald [32,33] enables the bonding between an aryl halide and an amine (Figure 2c). After the activation of the precatalyst and the oxidative addition of the aryl halide on the palladium center, an amine addition leads to a bond between the nitrogen and the metal center. Then, deprotonation induced by a base eliminates the halogenated compound. Finally, a reductive elimination forms the C–N bonding between the amine and the first aryl, generating the target molecule while returning the catalyst into a zero-oxidation state for a new cycle.

### 1.3. Life Cycle of a Suzuki’s Catalyst

Catalysts are often considered compounds that are not consumed by the reaction and return to their original form at the end of synthesis. However, the chemical reality is quite different since the catalysts—and especially the homogeneous ones—degrade over time. In this section, the degradation and regeneration of catalysts will be explored through the example of a Suzuki coupling.

#### 1.3.1. Suzuki’s Catalysts

In their first experiments, N. Miyaura et al. [21] used catalysts with a palladium center and phosphine ligands such as the bis(triphenylphosphine)palladium(II) dichloride—abbreviated in PdCl_2_(PPh_3_)_2_. Indeed, phosphine ligands imply a square–planar structure around the palladium center, making the metal accessible on both sides of the horizontal plane and facilitating oxidative additions. Nevertheless, Suzuki cross couplings can be performed with other platinoids such as rhodium [34] or ruthenium [35]. Recent research used other transition metals as homogeneous catalysts, such as nickel which is an interesting substitute in terms of its availability and inferior toxicity [36,37]. However, PGMs are still mostly used thanks to their good selectivity and their use with mild operating conditions [38]. Another approach under development focuses on cross couplings in aqueous media with the use of a hydrophilic catalysts [39]. J. A. Weeden et al. [40] used a complex between palladium acetate (Pd(OAc)_2_) and water-soluble 1,3,5-triaza-7-phosphaadamantante ligands (PTA) for a Suzuki coupling in a water/acetonitrile media. Moderate reaction yields between 36 and 66% were obtained with an operating temperature of at least 80 °C for enabling the activity of the catalyst. Thus, an important degradation of the catalyst into black palladium was observed. Aqueous cross couplings are therefore still challenging in many ways and hydrophilic catalysts are not easily commercially available. Finally, an emergence of heterogeneous catalytic processes for cross-coupling reactions has been observed over the years [41]. Heterogeneous catalysts form an interesting alternative thanks to their good recoverability and stability towards oxidation. A. Papp et al. [42] used palladium grafted on silica particles (Pd-SiO_2_Ph) for Suzuki reactions at 100 °C leading to reaction yields between 35 and 91% depending on the reagents. The disadvantages of heterogeneous catalysts compared to homogeneous ones are the lower reaction yield and selectivity and the less gentle working conditions. For these reasons, chemical industries still tend to avoid the systematic use of heterogeneous catalysts [43].

#### 1.3.2. Catalyst Degradation

**Figure 3:**
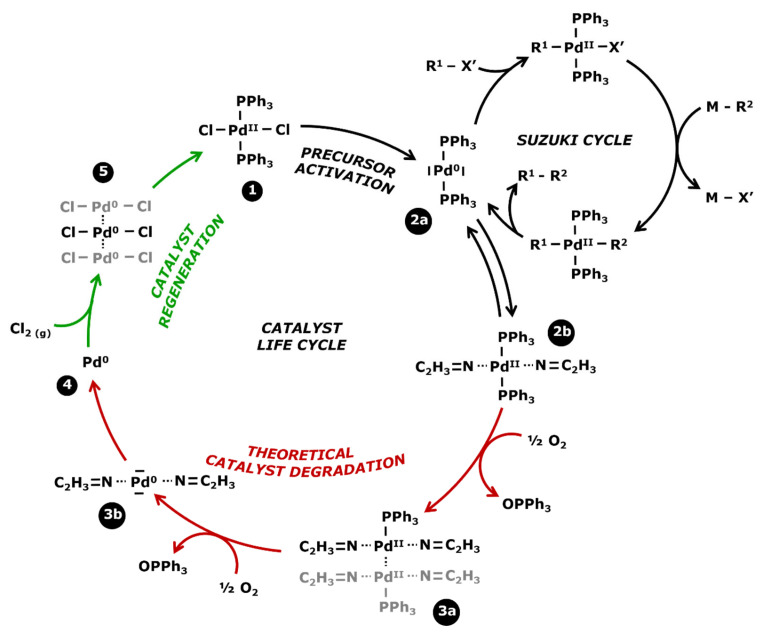
Life cycle for a Suzuki’s catalyst: reaction, degradation, and regeneration.

Homogeneous catalysts tend to degrade at the end of the reaction in the absence of reagents. In order to be easy to understand, Figure 3 presents the life cycle of the PdCl_2_(PPh_3_)_2_ catalyst with its use in a Suzuki cross coupling, its theoretical degradation process, and its regeneration which will be discussed in the next subsection.

Catalysts are commercially available in the form of a stable precursor or precatalyst (Figure 3—step 1) which needs to be activated for initiating the catalytic cycle. The active catalyst is a soluble molecule with a metal center at a zero-oxidation state (step 2a) and is recognizable by the yellow coloration of the effluents. According to W. L. Chul et al. [44], this color is typical of the triphosphate ligands linked to the metal center, with an infrared absorption band between 1629 and 1314 cm^−1^. However, palladium is stable at an oxidation state 2 which leads to the oxidative addition and the transmetalation of the Suzuki coupling. In the absence of reagents, the zero-oxidation state palladium center can bond to some solvent for regaining an oxidation state 2 (step 2b). Acetonitrile (ACN) is a good stabilizer, as observed by S. A. Cotton [45], but the bond is not strong enough to assure the long-term stability of the complex.

Homogeneous catalysts are well known to degrade themselves, mostly at the end of the reaction due to their impossibility to stay at the stable oxidation state 2. It eventually leads to the formation of metal palladium (step 4)—commonly called black palladium—which precipitates in the medium. The whole degradation process is not precisely known but various observations have led to an explanation. In 2006, H. Wong et al. [46] studied the separation of catalysts/products by a process coupling liquid–liquid extraction and nanofiltration. A decrease in the retention of palladium was found over time and correlated with the increasing contamination of palladium in the permeate. These results concluded with a degradation of the catalyst into smaller fragments that would more easily cross the membrane. The ratio between inactive soluble catalysts and active catalysts could not be determined because the analyses did not allow a differentiation of the oxidation state of the complexes. Therefore, the quantity of deactivated catalysts was estimated from the losses found in the permeate. M. Janssen et al. [47] observed a similar phenomenon in 2010, noticing a gradual decrease in the catalytic activity whereas the palladium concentration remained constant in the medium according to the analyses. It was concluded that some bonds inside the catalytic complexes were broken, resulting in the formation of fragments that were inactive but still soluble. W. L. Chul et al. [44] developed a degradation process of catalysts while studying the oxidation of Pd(OAc)_2_(PPh_3_)_2_ in contact with air. Monitoring the reaction by infrared, they observed the appearance of an absorption band at 1580–1411 cm^−1^ which characterized the oxidation of the triphenylphosphine ligands (PPh_3_) into OPPh_3_. Visually, the medium changed from yellow to orange. It was concluded that the molecules had reorganized themselves for forming [Pd(OAc)_2_PPh_3_]_2_ complexes where the palladium centers could reach an oxidation state 2. When the oxidation was carried on, the loss of a new triphenylphosphine ligand caused a brown coloration and then the precipitation of black palladium. Similar observations have been made by M. Gerlach et al. [48] who monitored the oxidation of ligands from a rhodium catalyst by infrared technology. With that mechanism in mind, in Figure 3 it was possible to theoretically determine the degradation process of the PdCl_2_(PPh_3_)_2_. At first, oxidation leads to the loss of one ligand and a rearrangement in complexes with two palladium centers bonding together (step 3a). The oxidation of the second ligand leads to an unstable complex (step 3b) and the precipitation of the black palladium (step 4). Moreover, this process is autocatalyzed: the presence of already-formed black palladium accelerates the degradation of what remains of the active catalyst.

#### 1.3.3. Catalyst Regeneration

**Figure 4:**
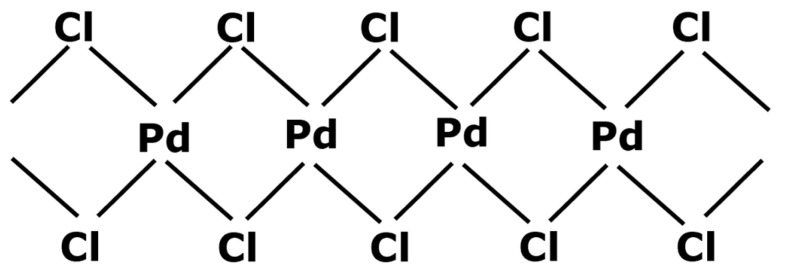
Stability of PdCl_2_ with an α-PdCl_2_ chain conformation.

Black palladium can potentially be regenerated into a catalyst precursor. S.A. Cotton [45] indicated two ways for forming palladium chloride (PdCl_2_) from metal palladium. The first one consists of dissolving the solid in aqua regia in the presence of chlorine gas. Aqua regia is a mixture composed of concentrated hydrochloric acid and smoking nitric acid. The second process involves heating the palladium up to 500 °C, still in the presence of chlorine gas, without exceeding 600 °C which is the decomposition temperature of PdCl_2_. This process is similar to the regeneration of other platinum group metals [49]. The resulting palladium chloride is a stable compound. Indeed, PdCl_2_ molecules stabilize themselves by taking the conformation presented in Figure 4 where palladium centers have an oxidation state 2.

Palladium chloride is a powder that can be kept without any major degradation and therefore can easily be found in commerce. However, this regeneration step of PdCl_2_ from black palladium leads to significant difficulty of industrialization, especially from a health, safety, and environment (HSE) point of view, with the constraints related to the storage and use at high temperatures of chlorine gas which is a particularly harmful compound.

However, the second regeneration step of the catalyst is quite simple. According to S. A. Cotton [45], PdCl_2_ has first to be dissolved in anhydrous acetonitrile and refluxed. As presented before, the PdCl_2_ molecules in solution for stabilization again bind with acetonitrile (abbreviated to ACN) and form a complex, PdCl_2_(ACN)_2_, which precipitates when the media is concentrated. The adding of light petroleum facilitates a complete precipitation. Then, once isolated, this new powder must be dissolved again. For forming the catalyst PdCl_2_(PPh_3_)_2_, S.A. Cotton [45] put the complex PdCl_2_(ACN)_2_ into acetone. The tri-1-phenylphosphine ligands were separately dissolved in acetone before being progressively added into the PdCl_2_(ACN)_2_ solution without any heating. The bond between palladium and acetonitrile is not very strong and, therefore, acetonitrile was replaced by the tri-1-phenylphosphine ligands with a yield of almost 100%. After the end of the addition and 1 h of agitation, ethyl ether was used for precipitating the newly formed PdCl_2_(PPh_3_)_2_. The solvent could then be eliminated to recover a precatalyst with great purity.

#### 1.3.4. Benefits of Catalyst Recovery

Platinoid-based catalysts have an important buying cost due to the sell price of the metal and the complexity of their generation process. As homogeneous catalysts, they often degrade over time [44], implying the constant need for industries to buy fresh ones or to rent them to a catalyst supplier such as Johnson Matthey. This second option consists of buying for a time of use with a supplementary compensation for metal loss. The toxicity of PGM-based catalysts to both humans and the environment [10,12] imply some constraints and the need for removal to avoid contamination of the synthesized molecules. In the pharmaceutical industry and an advanced treatment of platinoids is part of the recommended quality measures. The International Council for Harmonization of Technical Requirements for Pharmaceuticals for Human Use (ICH) [50] elaborates guidelines for encouraging worldwide industrial progress and protection of public health through various lines of work. Among them, the Guideline for Elemental Impurities ICH Q3D(R2) [51] focuses on risk assessment and treatment of residual traces for some hazardous elements. The PGMs are classified as 2B, meaning they represent a point of awareness if intentionally added during the production of APIs or intermediates which is the case when catalysts are used. Thus, the industry must implement purification steps contributing to extend the complexity of the whole process and the cost price of the syntheses. In addition, there is the need to retreat the waste effluents containing used catalysts due to the scarcity of rare metals [13]. Modelling of PGMs’ mining and consumption establishes an extraction peak in 2035–2050 followed by a decline of the resources [52], highlighting the necessity to work on metal recovery. In 2018, almost 91,000 kg of palladium were obtained through recovery—mostly from catalytic converters which is the main consumer industry [53]. In addition to this secondary supply, around 196,000 kg was needed, leading to an overexploitation of the mining resources. Moreover, the complexity of treatment processes of platinoids leads to an external destruction of waste containing metals by a third company with additional costs for the pharmaceutical production site. Thus, catalyst recovery and reuse in new chemical cycles could have an enormous economic impact and contribute to simplify and relieve the retreatment cycle. From a chemical point a view, homogeneous platinoid-based catalysts provide a great selectivity during syntheses [38], meeting the goals of a green and responsible chemistry where the number of chemical steps is reduced; the by-products are as limited as possible. Thus, the number of processes involving theses catalysts would increase in the coming years which would accentuate these challenges. Cross-coupling reactions are quite commonly used in the pharmaceutical industry [3,4]; however, quite often, these ways are abandoned in the early development phase in favor of a less efficient but less expensive process. Developing catalyst recovery and reuse could therefore make it possible to more regularly use these platinoid catalysts in production processes and lead to simpler and more efficient syntheses while respecting the economic and environmental goals of an industry.

## 2. Treatment Processes for Rare Metal Recovery

The literature reports several treatment processes for rare metals. However, not all of them can be adapted to pharmaceutical media which often imply complex matrices with organic solvents and in the presence of sensitive APIs. In addition, some treatment processes cannot directly deal with metals as catalytic complexes while others make their reuse as active catalysts impossible. This section presents treatment processes with their advantages and drawbacks for active catalysts recovery. However, the membrane processes will not be included in this section but will be the topic of the next one.

### 2.1. Heterogeneous Catalysts

Even if this review is focused on homogeneous catalysts, it is important to be aware that heterogeneous catalysts can also be performed for cross-coupling reactions. As the catalysts are supported on solid particles and present a better resistance towards degradation [54], the recovery and reuse of heterogeneous catalysts are not as challenging as homogeneous ones. However, some improvements are still being made in this field. Particles containing magnetite are rather innovative because they allow very easy separation thanks to their magnetic properties [55,56]. A.V. Dubey and A.V. Kumar [57] designed nanoparticles of FeO_3_ magnetite with a polydopamine layer on which palladium was adsorbed. These particles were active in Suzuki couplings and successfully reused up to five successive cycles. The reaction, performed at 80 °C, reached between 57 to 98% efficiency depending on the type of solvent or base used. Another interesting development is the use of bio-based materials for supported catalysts such as chitosan [58] or starch [59]. These green materials are accessible and at an affordable price which can be quite interesting for lowering the buying costs. However, the use of heterogeneous catalysts cannot systematically be the preferential choice for all pharmaceutical syntheses. Indeed, heterogeneous catalysts lead to less selectivity which can often be quite limiting when the API has a very high added value. In addition, operating conditions with heterogeneous catalysts are generally less mild, with temperatures as high as 80 °C for A. V. Dubey and A. V. Kumar [57] and 100 °C for the previously cited heterogeneous catalysis of A. Papp et al. [42]. Molecules of pharmaceutical interest can sometimes be heat-sensitive which is another limitation for the use of supported catalysts in some syntheses of this industry.

### 2.2. Adsorption Processes

**Figure 5:**
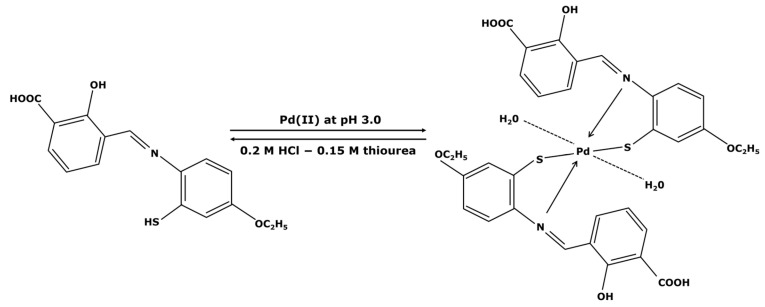
Adsorption/desorption mechanism of Pd^2+^ ions on a ligand developed by R. Awual et al. [60].

A well-known treatment process for metal removal from industrial waste is adsorption thanks to the good selectivity of sorbents towards metallic compounds. The mechanisms of adsorption are quite similar to the synthesis of supported catalysts with the palladium center chelated by the sorbent (Figure 5).

R. Awual et al. [60] synthesized a specific ligand for Pd(II) recovery with a maximum adsorption capacity of 164.20 mg g^−1^. After that, the adsorbent was simply removed by filtration and desorbed the palladium under the form of Pd^2+^ ions by addition of thiourea. The desorbed ligands could then be reused up to 10 cycles without a significant loss in adsorption capacity. Many types of adsorbents exist such as carbon-based materials [61] or silica gels [62]. S. Sharma et al. [63] compared various sorbents and indicated that silica gels and silica alumina gels have a relatively low cost, good thermal properties, mechanical stability, and good performance when it comes to capturing catalysts. Just like heterogeneous catalysts, magnetically recoverable sorbents are under development [64]. Biosorbents—made from low-cost green compounds such as chitosan [65], cellulose [66] or kraft lignin [67]—are also quite popular. S. Sharma and N. Rajesh [68] conducted laboratory tests with a biosorbent based on chitosan and β-Cyclodextrin. The result was a material with a maximum adsorbent capacity of 202 mg g^−1^ and a proven Pd-selectivity for a solution containing various ions. This biosorbent had been successfully tested to extract palladium grafted onto activated charcoal used as an industrial catalyst. However, for being efficient, the particles have to first be dissolved in an acidic solution for separating the activated charcoal and form an H_2_PdCl_4_ complex which was then adsorbed on the biosorbent with a 91% efficiency. However, S. Sharma and N. Rajesh [68] as well as R. Awual et al. [60] offered ways to recover the catalyst but without the possibility to reuse it directly. Indeed, the capture is carried out on the Pd^2+^ ions in acidic solution and it is therefore necessary to deactivate the catalyst by breaking the molecule. This leads to a need for catalyst regeneration which can be complex to implement, as previously discussed. These adsorbents also have the disadvantage of being pH-sensitive which imposed operating conditions for optimal treatment such as an acidic medium at pH 2 or 3. For pharmaceutical media, such acidity can cause some problems due to the potential sensitivity of the active ingredients. Nevertheless, some absorbents are directly able to treat catalysts under their active form. Commercially, silica adsorbent SiliaMetS^®^ Thiol from the company SiliCycles (Quebec City, QC, Canada) was successfully used to remove almost all of the 0.134 kg of [BrPdP(tBu)_3_]_2_ catalysts from a reaction media containing synthesized molecules [69]. After a 82-h stirring at 50 °C in the presence of 1.458 kg of this industrial adsorbent and a filtration step for removing SiliaMetS^®^, only 1–2 ppm of palladium was left in the organic matrix. A. Stumpf et al. [70] also used industrial SiliaMteS^®^ Thiol to successfully remove Pd catalysts from a reaction medium. After 3 h of stirring at 90 °C, the palladium amount decreased from 2400 ppm to 3 ppm. Only 20% *w*/*w* of sorbents were used, leading to an experimental adsorbent capacity of 200 mg g^−1^. Under the form of particles, adsorbents can be used in bulk or in cartridges. In bulk, the sorbents are suspended inside the reaction medium in an agitated reactor. After a defined contact time, the particles must be filtered from the mixture. Instead of this process, the sorbents can be packed in a cartridge which removes the filtration step and offers an improved exchange between sorbents and catalysts [71]. Successive layers of different sorbents can also be implemented for improving metal elimination. Adsorbents can also be grafted on resins. According to J. C. Lee et al. [72], resins can be used as ion exchangers. Just like other sorbents, the process is divided into steps: adsorption of the metal ions and then desorption by adding an eluent which is often an acidic solution. In order to adsorb, the resin has to first be functionalized with groups that can be selective towards a specific metal. P. Moleko-Boyce [73] used triethylenetetramine (TETA) for capturing palladium with a loading capacity of 192.0 mmol g^−1^. However, there are two major drawbacks to adsorbent use. First, the previously presented study of A. Stumpf et al. [70] described a small batch of 3.87 kg scale. In the case of bigger batches, the needed quantity of catalysts could threaten the economic viability of such a process by increasing too much the adsorbent consumption. Then, it is quite challenging to obtain a functional desorbed homogeneous catalyst after an adsorption process. For the previously cited industrial silica sorbents, two ways are usually carried out for recovering isolated metal: pyrometallurgy which consists of burning the whole sorbent–catalyst complex and leads to metal solids and hydrometallurgy which consists of dissolving the complex in an acidic solution and leads to metal ions. These two processes break the catalyst’s inner bonds and therefore deactivate it. Therefore, adsorbents can be a good way for industries to remove residual traces of a metallic catalyst but may not be yet adapted for direct catalyst reuse in new chemical cycles. It is why industrial absorbents are commonly sold under the name of a “metal scavenger”.

### 2.3. Liquid–Liquid Extractions

Liquid–liquid extractions are also well known for separating homogeneous impurities in a medium. Some approaches can be found in the literature. Ionic liquids are quite commonly used for treating metal ions [74]. Ionic liquids are salts possessing a low fusion point. M. Rzelewska-Piekut and M. Regel-Rosocka [75] used quaternary phosphonium salts for selectively removing palladium, platinum, rhodium, and ruthenium ions present in a synthetic mixture. When soluble, ionic liquids bond to the metal centers and form a complex that precipitate once the medium is cooled. Then, they can be easily eliminated. Finally, the metal ions can be recovered by stripping with a solution of acid and thiourea. In another approach, B. Gupta [76] and A. P. Paiva [77], respectively, used Cyanex^®^ 923 (Compton, UK) and Cyanex^®^ 471X—industrial extractors—to move palladium ions from an aqueous phase to a toluene phase. Just like ionic liquids, Pd^2+^ ions can be recovered by stripping with an acidic thiourea solution. However, most platinoid catalysts are solvent-soluble and need to be separated from an organic phase. For the pharmaceutical industry, this organic phase also usually contains the active ingredient which induces some constraint for preserving the stability of the molecule. The choice often made by industries for removing catalysts is to use chelating agents such as ethylenediamine (EDA) and its derivatives [78]. Chelating agents are polydentate ligands, meaning they bond several times on the metal center. As a bidentate ligand, ethylenediamine bonds twice. The efficiency of the chelation process is due to the ring formation that is more stable than the previous catalytic complex [79]. Ethylenediamine is water-soluble and thus, after the chelation process, the catalyst can easily be removed from an organic phase and dragged into an aqueous phase. Several chelating agents are known, the most common being the ethylenediamine derivatives like ethylenediaminetetraacetic acid (EDTA) or hydroxyethylethylenediamine [79]. However, in addition to a phase change, chelation often inhibits the activity of catalysts and leads to a need for retreatment process. This alternative could be useful for removing catalysts from a pharmaceutical medium but does not permit their reuse in new chemical cycles. For keeping the catalyst under its active form while separating it from the synthesized molecule, it is therefore necessary to implement a liquid–liquid extraction from an organic phase to another non-soluble organic phase. Thermomorphic properties of solvents could be useful to reach that objective. J. M. Dreimann et al. [80] studied an extraction with dimethylformamide (DMF) and n-decane. When heated, the medium was homogeneous and the chemical reaction was achieved in this matrix. The reaction was a hydroformylation in the presence of a rhodium catalyst. At the end of the reaction, the mixture was cooled and became biphasic thanks to the thermomorphic properties of the two solvents. The DMF phase containing only catalysts was directly re-injected into the reactor while the n-decane phase contained the entirety of the synthesized molecules and some residual catalytic complexes. This kind of process can lead to the separation and reuse of at least a part of the catalysts. However, it cannot be systematically applied and depends a lot on the characteristics of the solvents used in the chemical reaction.

### 2.4. Catalysts’ Intrinsic Properties

Sometimes, the intrinsic properties of catalysts can enable the separation of the catalyst from the rest of the medium without the necessity to add an extractant. Tessema et al. [81] synthesized a palladium-based complex [PdCl_2_ [5,5′-bis-(R_f_CH_2_OCH_2_)-2,2′-bpy] and used it for Suzuki and Sonogashira cross couplings. This specific catalyst had thermomorphic properties and it was thus homogeneous at 140–145 °C during the reactions and became a heterogeneous catalyst when the media was cooled down. Then, the catalysts were recovered by a simple filtration step before being reused up to eight successive cycles for the study of the Sonogashira reaction. However, not all catalysts possess thermomorphic properties and the harsh operating conditions cannot always be adapted to all pharmaceutical syntheses, reducing the opportunities for such a process. Nevertheless, it is worth noticing that some interest can appear for catalyst recovery in a few very specific cases.

### 2.5. Electrochemical Processes

Electrochemical processes can be an alternative for metal recovery. Electrolysis is an interesting process inducing the precipitation of metal particles [82]. Then, all the metal can be recovered from the medium by performing a simple solid–liquid filtration. V. K. Varentsov and V. I. Varentsova [83] used a carbon electrode to reduce and recover palladium from minerals previously dissolved in an acidic solution. It seems possible to adapt this process at an industrial scale. N. Warner and M.L. Free [84] described a recycling process for dissolved palladium using a copper cathode with a large exchange area that could collect up to 1 kg of metal. With an electric current of 10 amperes and a gradually increasing voltage, it was possible to recover 90% of the palladium on the surface of the cathode in 10 h and in 20 h with a 5-ampere current. This recovery system called Gold Bug could treat concentrated solutions up to 5 ppm while having a low-energy cost compared to ion exchange resins. However, since metal selectivity is a complicated parameter, these tests were carried out with media containing only palladium. The quantity of metal treated can also be problematic. Indeed, electrolysis is said to remove some ppm whereas cross-coupling reactions are often carried out with much more catalysts. In a review, C. S. Horbaczewskyj and J. S. Fairlamb [85] identified the quantity of catalysts used for cross-coupling reactions and expressed it in mole percentage (mol%) via a concentration ratio between the quantities of catalyst towards the limiting reagent used for the synthesis. For Suzuki cycles, the chemistry is carried out in the presence of 0.1 to 5.0 mol% of palladium. In addition, electrolysis deals with palladium under an ionic form and therefore is confronted with the need to voluntarily degrade the catalyst and implement a regeneration process of the complex. Thus, electrolysis does not seem to be adapted for the metal recovery of catalysts in pharmaceutical media nor for reuse of these catalysts in new chemical cycles.

There are also electrochemical membrane processes—such as electrodialysis—which enable the transfer of salts thanks to an electric field and the use of anionic and cationic membranes. J. W. Blackburn [86] recovered rhodium-based catalysts in methanol using this process. He indicated that electrodialysis has the advantage of not requiring large pH adjustments compared to what is needed for many adsorbents. In addition, there is no need for complementary crystallization and/or filtration processes. Another electrochemical membrane process is capacitive deionization. D. Kim et al. [87] managed to retain 99% of Pd^2+^ ions in the concentrate after several cycles of use, starting from solutions loaded at 10 and 100 mg L^−1^. However, this technology is not applicable for pharmaceutical media because capacitive deionization is only performed in aqueous media whereas most catalysts are solvent-soluble. Thus, these processes can be used for metal recovery but do not permit the direct reuse of active catalysts. In addition, electrochemical processes are confronted with some challenges for expanding to an industrial scale. Most studies on metal recovery only focus on laboratory experiments without necessarily seeking to extrapolate them for a larger process. An industrial scale-up implies some challenges in treating high volumes of effluents. Working with several units in parallel or in series could be a solution but it will lead to important rises in investment and operating costs [88]. Moreover, the need for an ATEX environment could lead to additional problems towards electrochemical processes.

### 2.6. Comparison of Treatment Processes

A large range of processes describe treatments with the objective of platinoid recovery. However, not all of them can lead to the isolation of active soluble catalysts and enable their reuse in new chemical cycles. Table 2 summarizes the previously presented processes with the type of recovery that can be reached.

Most processes found in the literature operate by capturing metallic ions after preliminary dissolution of the compounds into an acidic solution. Therefore, it could only lead to metal recovery and does not permit the isolation of active soluble catalysts and their recycling in new chemical reactions. This gathers various sorbents [60,63,68] as well as the electrochemical processes [83,86,87].

The second main option could directly be implemented for treating catalytic complexes in their organic matrixes via a chelation processes. It regroups some other sorbents [69,70] and most of the liquid–liquid extractions which are induced by a chelating agent [76,77]. These processes are easier to implement as they do not need preliminary conditioning of the compounds. However, the desorption process is destructive towards the chelated complexes whether it is performed by pyrometallurgy (burning) or hydrometallurgy (dissolving into an acid). Thus, every treatment process related to chelation mechanisms cannot permit the recovery of platinoids under an active catalytic form.

The only way to reach a homogeneous catalyst recycling seems to be case specific [80,81]. Some examples of liquid–liquid extractions without the addition of a chelating agent can be found in the literature. The major issue of pharmaceutical media is the presence of APIs or intermediates with solubility properties often similar to the catalysts used. Reaching an effective gap of solubility factors between the two compounds without adding of a chelating agent is therefore relatively hard to reach and limited to a few examples dependent on the compounds and/or the matrix involved. Thus, an efficient and generalized recovery of recycling of homogeneous platinoid catalysts from pharmaceuticals seems to be quite challenging.

## 3. State-of-the-Art Catalyst Recovery by Membrane Processes

Membrane processes are another large family of treatment processes with great potential regarding the problems presented in this review. This section is focusing on membrane processes and their perspectives for separating APIs from catalysts in a reaction medium and reusing these metallic complexes while monitoring their degradations.

### 3.1. Membrane Processes

Several well-known membrane processes can be considered for catalyst recovery. Ultrafiltration enables steric separation depending on the pore size of the membrane material. Due to the small size of the catalysts, ultrafiltration membranes are not commonly used for this specific problem. Nevertheless, some studies focused on isolating catalysts or ions stabilized by micellar systems [89,90]. This process is known as micellar-enhanced ultrafiltration (MEUF). Complexation by a polymer is also possible but the studies are limited to the recovery of metal ions in aqueous media [91]. Due to the small sizes of catalysts, organic solvent nanofiltration (OSN) is the main applied technology [92,93]. With nanofiltration membranes, the cutoff point is not defined by a pore size but is reported as a molecular weight cut off (MWCO). B. Xaba et al. [94] studied the recovery of a PdCl_2_(PPh_3_)_2_ catalyst used for a Heck reaction. Nanofiltration membranes were compared with different MWCO as well as different solvent matrixes. The catalyst/product separation was performed by a simple size difference: the small product with a molecular weight of 180 g mol^−1^ was passed into the permeate while the catalyst, larger and with a weight of 701.9 g mol^−1^, was partially retained by the membrane. Thus, membrane processes can enable the isolation of a catalyst from the reaction media if the size difference between the compounds present in the matrix permits it. However, in nanofiltration, the separation is not only determined by steric effects. Indeed, some charge effects can appear and have an impact on retention behavior. A. Keraani et al. [95]—while studying the nanofiltration of catalysts weighted by large aromatic groups—found that the biggest catalysts were not the more easily retained due to chemical interactions with the membrane material. Reverse osmosis (RO) is another common membrane process often used in water treatment for desalination and/or salt concentration. In this case, dense membranes are used and the transfer through them follows a model of solution diffusion. Recent studies explored the possibility of treating organic media by performing organic solvent reverse osmosis (OSRO). However, these searches mainly focus on solvent purification from binary mixtures without the presence of salts [96,97]. A few studies also focus on metal removal from wastewater. Heavy metals such as Cu, Cr, or Ni can be successfully retained under their ionic forms [98] but the recovery of homogeneous catalysts with reverse osmosis membranes is quite recent and is still focused on mild organic solvents such as acetonitrile or 2-propanol [94]. However, forward osmosis (FO) seems to have some potential for catalyst recovery. Y. Cui and T. S. Chung [99] worked on the concentration of APIs from an organic matrix. According to them, forward osmosis does not apply any pressure and therefore limits the risks of fouling the membrane or degrading the sensitive APIs. In this study, the medium composed of ethanol was concentrated thanks to the use of lithium chloride as a draw solution on the other side of the membrane. Organic solvent forward osmosis has not yet been used for separating catalysts and the searches are still focused on the laboratory scale. Thus, an application for catalyst recovery and reuse in industries cannot be implemented yet.

### 3.2. Membrane Materials

**Figure 6:**
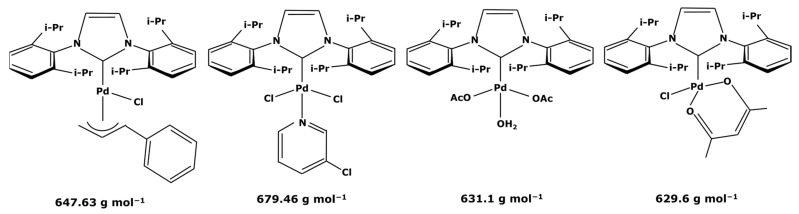
Catalysts derived from Pd(OAc)_2_ used by D. Ormerod et al. [107].

Membranes are generally regrouped depending on their materials. Polymeric, ceramic, and hybrid membranes can be found with different advantages and drawbacks for each of them. Polymeric membranes have the great advantage of being adapted to all MWCO, facilitating the separation between two compounds such as a catalyst and pharmaceutical molecule. On the contrary, it is quite difficult to find mineral membranes with very low MWCO at an industrial scale. However, the stability of polymeric membranes towards solvents is still challenging and often leads to membrane deterioration [100]. Hybrid membranes have a composite structure. Due to their properties, some of them can be solvent-resistant [101,102] and therefore useful for catalyst recovery from organic media. N.S.A. Razak et al. [103] used a STARMEM 240 polyimide membrane for retaining an HRh(CO)(PPh_3_)_3_ rhodium catalyst used for a hydroformylation reaction in toluene. The metallic complex of 400 Da had a sufficient size difference with the synthesized products for being separated by the membrane. A 93% retention rate of the catalyst was achieved with a suitable flow by working at 20 bar pressure. However, the polymeric part of this hybrid membrane could produce chemical interactions with compounds and thus disrupt the separation. L. White [104] observed a favored transport for compounds containing aromatic nuclei which limited the efficiency of their retention when polyimide membranes were used. In addition, hybrid membranes are still unstable towards harsh solvents such as dichloromethane (DCM) or dimethylformamide (DMF) [100] which can be used as matrixes for chemical reactions in the pharmaceutical field. Nevertheless, the improvement of solvent stability by designing new materials is currently under study [105,106]. Finally, unlike polymeric or hybrid membranes, ceramic possesses solvent-resistant properties and can therefore be quite interesting as a membrane material for the pharmaceutical industry. Ceramic membranes can also resist strong washing conditions and are easily adaptable to an industrial scale. D. Ormerod et al. [107] used ceramic membranes for separating palladium catalysts from the synthesized products of a Suzuki reaction in organic media. Ceramic membranes are hydrophilic and therefore less efficient with non-polar solvents so the membrane surface was modified for making it amphiphilic and reaching up to 99% rejection rate depending on the studied complex and the type of process. The tested catalysts were palladium (II) acetate derivatives like Pd(OAc)_2_ previously modified by adding ligands. These newly formed catalysts are presented in Figure 6.

The major limitation of ceramic membranes is their MWCO. Due to the material structure, it is much more difficult to reach a very low MWCO during the conception of the membrane compared to polymeric ones. Therefore, the minimum MWCO for ceramic membranes commercially available at the industrial scale is 1 kDa, meaning it enables a 90% retention of compounds with a molecular weight of 1000 g mol^−1^. However, these limitations tend to disappear as new ceramic membranes with an MWCO of around 450 Da or even smaller become more common commercially, such as those developed by Inopor^®^ [108].

### 3.3. Catalyst Enlargement

The separation between catalysts and APIs is not always that simple in the pharmaceutical field. Indeed, active pharmaceutical ingredients can often have a similar size to the catalytic complex of around 400–800 g mol^−1^. For facilitating the separation, a potential approach consists of changing the size of the catalyst to improve its retention. As presented before, ceramic membranes offer perspectives for catalyst recovery in organic media but are limited by their MWCO. For avoiding this limitation, some work on catalyst enlargement has been made. A. Keraani et al. [95] studied the recovery by nanofiltration of ruthenium catalysts whose molecular weights were previously modified by the addition of phenyl groups, substitutions by carbonated alkyl chains, and/or complexation of two or three metal centers. Thus, a panel of catalysts was obtained with molecular weights from 627 g mol^−1^—for the original commercially available complex—to 2195 g mol^−1^. The study concluded that optimal retention was achieved with 887 g mol^−1^ catalysts with a recovery rate of 92%. This catalyst was then engaged in five consecutive cycles of metathesis before observing a significant reduction in the catalytic activity [95]. The aim of this study was to maximize a low but already existing retention strategy of a catalyst: namely 65–70% for the commercial catalyst. Indeed, the metathesis resulted in the formation of molecules with much lower molecular weights of 251 and 223 g mol^−1^ for the reagent and the product, respectively. This approach is quite interesting because it confirms the interest of catalyst enlargement for facilitating recovery. However, adding or modifying ligands can influence the efficiency of the complex and even lead to its deactivation. Two parameters are therefore important to monitor. First, the steric hindrance of the complex increases with larger ligands. This could facilitate the membrane separation between catalysts and products but has an impact during the catalytic cycle. Reductive elimination is favored but the oxidative addition step is limited because of the accessibility around the metal center. This change may therefore result in a decreasing reaction yield or even the formation of a different synthesized molecule [109]. This strategy could be balanced by rising the quantity of catalysts used for the reaction but only if the recovery and reuse are enough to make it economically viable. The second factor to keep in mind during catalyst enlargement is the change in the electronic parameters of the complex which may or may not have a negative impact on catalytic activity depending on the changes [110,111]. Thus, catalyst enlargement can only be implemented in a process if the efficiency of the new catalyst has been studied. The rest of this subsection is focused on the several ways to reach catalyst enlargement.

#### 3.3.1. Use of Another Catalyst with Similar Activity

To have a heavier catalytic complex, it can be sufficient to find a new commercially available one that directly meets the criteria of an improved retention. However, catalysts with a high molecular weight are not common. According to the portfolio of homogeneous catalysts sold by Johnson Matthey [112], there is a small number of complexes with a molecular weight over 1000 g mol^−1^. None of them are recommended for cross-coupling reactions. Instead of looking for a heavier catalyst, it could be therefore interesting to search for complexes with high steric hindrance which could enable a sufficient rejection rate by the membrane. As discussed before, the activity of a catalyst with a high steric hindrance must be confirmed for each specific reaction. Some of these complexes are presented in Table 3.

**Figure 7:**
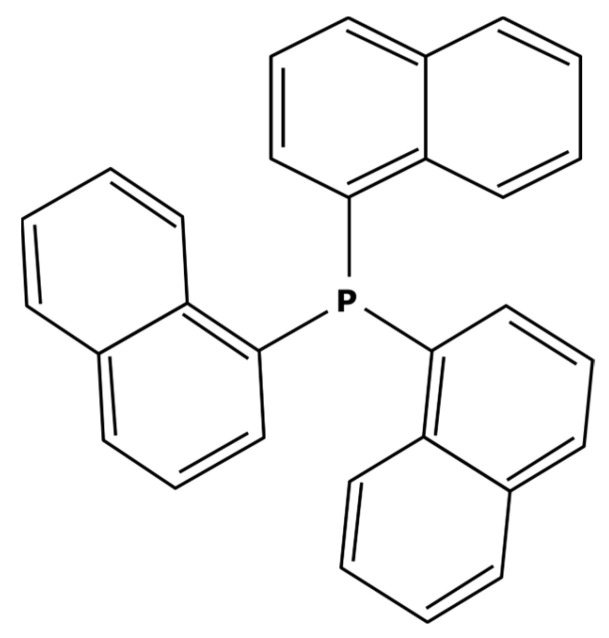
Tri-1-naphtylphosphine ligand.

These large commercially available catalysts have complex structures and thus their selling cost is much higher that traditional Suzuki catalysts such as PdCl_2_(PPh_3_)_2_. The use of this kind of catalyst can therefore be limited by an economic aspect. Even though platinoid-based catalysts with a molecular weight higher than 1000 g mol^−1^ are difficult to find, some of them can quite easily be synthesized in a laboratory. S.A. Cotton [45] described the synthesis of a catalyst from palladium chloride (PdCl_2_). As previously explained, the process consisted of putting PdCl_2_ and the desired ligands in solutions and adding one to the other without heating. For synthesizing a heavier catalyst than PdCl_2_(PPh_3_)_2_ while keeping the same activity, it is important to conserve a square–planar structure around the palladium center. With that objective in mind, aromatic substituents—which are flat by definition—can be good candidates. Moreover, these ligands will have electronic properties very similar to triphenylphosphine (PPh_3_) which should permit the same activity. Thus, tri-1-naphthylphosphine (PN_3_) presented in Figure 7 could be considered as a promising ligand.

Replacing the triphenylphosphine ligands with tri-1-naphthylphosphine would then enable the obtention of the PdCl_2_(PN_3_)_2_ complex which has a molecular weight of 1001 g mol^−1^ that could theoretically be better separated. It is worth noticing that this new catalyst has already been used in Sonogashira coupling reactions [113,114]. In addition, tri-1-naphthylphosphine is commercially available at a reasonable price from different chemical suppliers which can be quite interesting for industrial use.

#### 3.3.2. Ligand Modification Starting from a Known Catalyst

**Figure 8:**
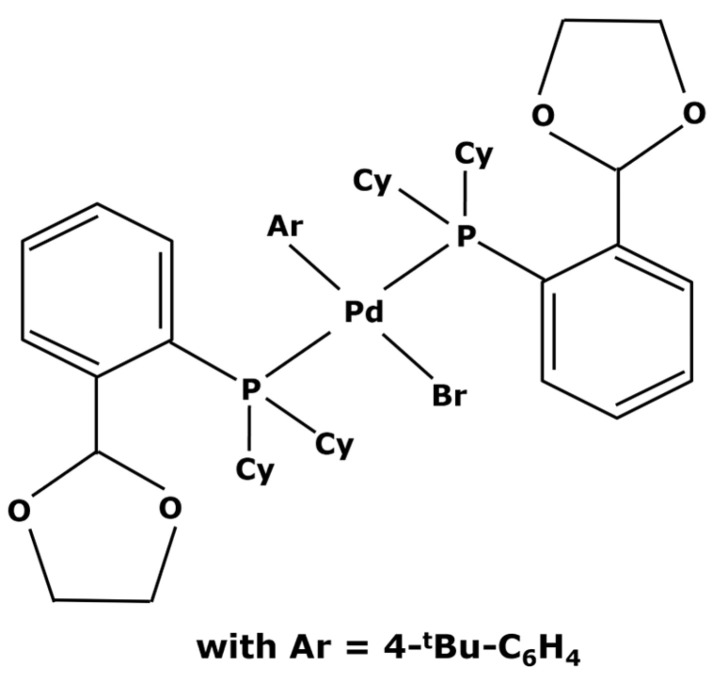
Catalyst synthesized by X. Bei et al. [117].

Instead of buying a completely new catalyst, it can sometimes be possible to perform a change in ligands. Starting from commercially available complexes, some modifications can be made by obtaining the desired characteristics without having to carry out a complete synthesis of a catalyst. Several studies are reported in the literature [115,116]. X. Bei et al. [117] used the easily affordable Pd(dba)_2_ and 4-tBu-C_6_H_4_Br. This led to the creation of the catalyst represented in Figure 8.

**Figure 9:**
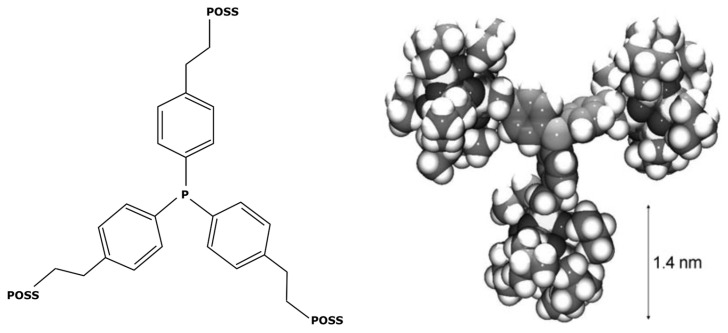
POSS-enlarged triphosphine ligands with its 3D model [47].

This new complex, with a molecular weight of 1000 g mol^−1^, was then used in Suzuki cycles leading to yields from 83 to 97% depending on the reagents and products of the reactions [117]. In other ways, M. Janssen et al. [47] weighted down triphenylphosphine ligands with polyhedral oligomeric silsesquionaxe (POSS) for obtaining heavier ligands, as shown in Figure 9.

In their study, these ligands were used for increasing the molecular weight of the RhCl_2_(PPh_3_)_2_ complex, reaching 5582 g mol^−1^. It permitted an easy separation from the rest of the medium after a hydroformylation reaction. POSS is often cited in the literature about the formation of insoluble nanoparticles. V. Somjit et al. [118] used POSS for designing heterogeneous palladium-based catalysts efficient for Suzuki and Heck reactions. However, according to Janssen et al. [47], their enlarged ligands remained soluble in the medium. This was also confirmed by A. Kajetanowicz et al. [119] who similarly used POSS for increasing the molecular weight of a catalyst, this time a ruthenium complex used in metathesis reactions. However, ligand modification does have some drawbacks. Each additional step in an industrial process generates increased resource demands, both human and technical; generates an extension of the production time; and requires the management of new waste effluents and eventual by-products. The synthesis described by X. Bei et al. [117] is composed of many steps: the synthesis of ligands, their purification by recrystallization, and the subsequent synthesis of the catalytic complex. Such a process could lead to many additional costs, limit the economic viability of industrial implementation, and worsen the environmental impact.

#### 3.3.3. Pincer Ligands

**Figure 10:**
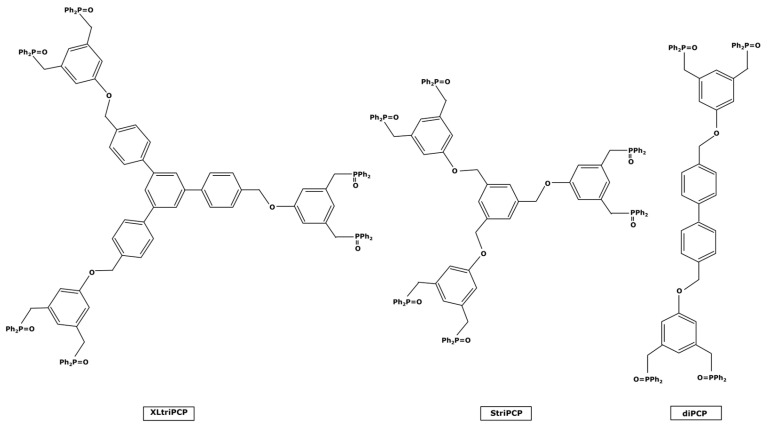
Heavy pincer ligands under their oxide-protected form.

Molecular weight enlargement can also be conducted thanks to pincer ligands. These molecules present two traditional ligands at their ends which bind to the metal center by forming a pincer-like structure, hence the name given to them. The bonding reaction is quite similar to the chelating process discussed for extractants or sorbents; in fact, pincer ligands are sometimes used as chelating extractants for metal ions before a liquid–liquid extraction step [120] or are structures for heterogenous-supported catalysts [121,122]. These pincer ligands are quite present in the literature and can be used as homogeneous catalytic complexes in organic solvents without inhibiting the catalytic activity [123]. However, they often have small sizes. H.M. Lee et al. [124,125] can be mentioned for their work on ligands for palladium catalysts used in Heck and Suzuki reactions. The resulting complexes had molecular weights around 500–600 g mol^−1^, too far from the target value of 1000 g mol^−1^ that could be interesting for a nanofiltration recovery. Nevertheless, N. J. Ronde et al. [126] synthesized the three pincer ligands presented in Figure 10, which may be of interest for catalyst enlargement.

The smallest of these—diPCP—could form a complex of 1294 g mol^−1^ if it was linked to a single PdCl_2_. However, these ligands were tested only in the case of allylic alkylation and allylic amination reactions [126] and it would therefore be necessary to check their activity on other catalyzed reactions to ensure that the steric hindrance caused by the bonding would not interfere during the additive oxidation step and therefore inhibit the reaction cycle.

#### 3.3.4. Support Molecules

**Figure 11:**
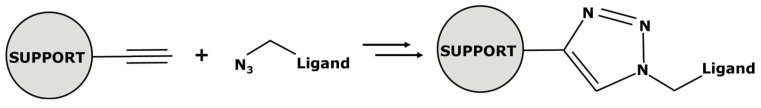
Mechanism of “click” chemistry.

Another approach to weight enlargement involves the use of a large support molecule—called a dendritic support—with traditional catalytic complexes at its ends. The ligands are attached to the support through “click” chemistry. This term refers to a quick and efficient cycloaddition in mild conditions (Figure 11).

**Figure 12:**
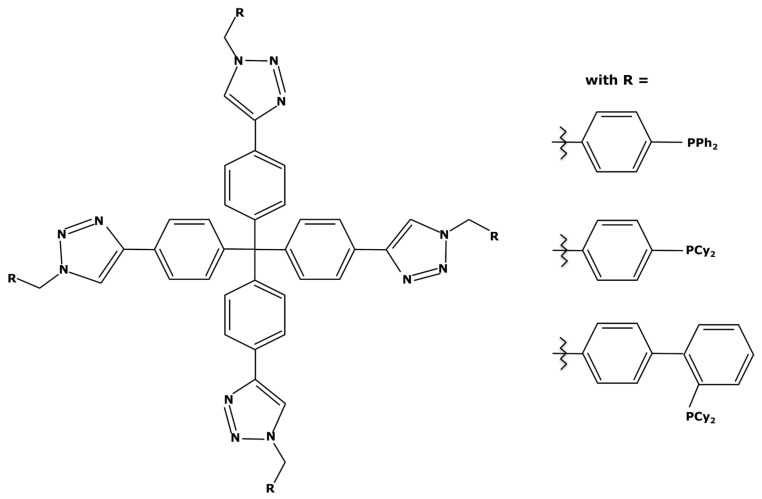
Support molecule with several traditional ligands at its ends.

“Click” reactions are often used for the heterogenization of homogeneous catalysts in order to improve their recovery [127]. However, M. Janssen et al. worked on immobilization on soluble dendritic supports. They addressed this topic in a general publication on techniques for catalyst enlargement [128] and in an article focusing on a Suzuki reaction with palladium [129]. This support molecule, once bound to a metal center, can be used effectively for up to five successive Suzuki cycles while being easily recoverable by nanofiltration with losses of less than 0.8% *w*/*w* at each cycle [129]. The representation of this support molecule is shown in Figure 12.

Considering this molecule with the triphenylphosphine ligands, the resulting complex could have a molecular weight of 1820 g mol^−1^ with a PdCl_2_ attached to it. However, this molecule is rather complex to synthesize and not commercially available which can make its implementation difficult at an industrial scale. In addition, like the use of pincer ligands, support molecules can negatively impact the oxidation addition during the catalytic cycle due to the high steric hindrance. It is therefore necessary to study the catalytic activity for each case.

#### 3.3.5. Nanomicellar Complexes

The last catalyst enlargement method presented in this review consists of trapping the complexes inside micelles. This process is called micellar enhanced ultrafiltration (MEUF). D. Schwarze et al. [130] studied the recovery of rhodium-based catalysts with this approach. Micelles were created from different types of surfactants—anionic, cationic, and non-ionic—and were used as catalysts for a hydrogenation reaction after being recovered by ultrafiltration. The retention of catalysts was equal to the retention of the micelles: up to 93% in the best configuration. However, it was noticed that the pore size of the different membranes tested had less impact on retention than the polarity. Thus, the filtration efficiency became more of a matter of interactions between the surfactant and the appropriate membrane [130]. This study was carried out in a relatively simple aqueous environment, much different from the organic environment of most pharmaceutical syntheses. Thus, it consists of heterogeneous catalysis in a biphasic environment with the drawbacks that it implies: less catalytic efficiency and selectivity and a need for harder operating conditions. In addition, if the reaction must be performed in an organic phase, the trapped catalyst inside the micellar system must be water-soluble. As previously discussed, this kind of catalyst is uncommon and expensive. In addition, the efficiency of the nanomicellar catalytic complexes is lower compared to a traditional catalytic reaction. MEUF processes enable a facilitated catalyst recovery but limit the reaction yield [131]. Thus, the use of micellar systems as an enlargement technology does not seem adapted for a pharmaceutical purpose.

### 3.4. Operation of a Membrane Process

One benefit of membrane processes is their adaptability to both batch and continuous processes. This can be quite useful for the pharmaceutical industry as both modes are implemented in production sites. For a continuous installation, a regular flow of reagents is introduced for the cyclic reaction while the products are treated by membrane processes and recovered in the permeate. Concerning the addition of catalysts, two configurations are possible: one with feed flows containing fresh catalysts and reagents and the other one without the fresh catalyst input. Continuous processes have several advantages compared to batch ones, the main one being the limitation of catalyst degradation which mainly appears in the absence of reagents due to the constant addition of fresh reagents. In addition, L. Peeva et al. [132] observed a better retention rate for a continuous process compared to its batch equivalent, resulting in lower catalyst contamination in the permeate. A Heck reaction was performed in a reactor/separator equipped with a nanofiltration membrane. The reagents were continuously injected into the reactor and the feed pressure drove the mixture through the dead-end filtration membrane. A continuous experiment for 1000 h resulted in twenty times less contamination of the synthesized products recovered in the permeate compared with the batch tests. D. Ormerod et al. [107] compared a batch process with the two continuous configurations for the same Suzuki reaction. According to the obtained results, the continuous configurations had many advantages such as a better control of the quantity and stability of the catalyst and therefore a better reaction yield. This was particularly observed in the presence of the fresh catalyst input. However, catalyst retention is best for the batch mode. Contamination of the product is increased during continuous experiments even if it remained at a very low level. This conclusion is not necessarily opposed to that of L. Peeva et al. [132] because the processes are very distinct, especially in terms of filtration: they are dead-end for L. Peeva et al. [132] and tangential for D. Ormerod et al. [107].

#### 3.4.1. Continuous Processes without Fresh Catalyst Input

The first continuous configuration consists of putting an initial quantity of catalyst in the reactor without any additional input through time. Thus, the reaction stops when the activity of the catalyst becomes too low due to its natural degradation over time. The reaction time is then limited to a value previously determined by experimentation. M. Janssen et al. [47] worked on an alkene hydroformylation reaction at a laboratory scale. Good retention and catalytic activity were achieved for two weeks before observing the deactivation of the rhodium catalyst.

#### 3.4.2. Continuous Processes with Fresh Catalyst Input

The other configuration consists of implementing a continuous input of fresh catalysts in the reactor. This makes it possible to balance the loss of activity over time. E. J. O’Neal and K. F. Jensen [133] had retention rates from 99 to 99.5%. They considered the loss of catalyst through the permeate as the deactivated quantity of catalytic complexes and compensated this loss with an equivalent addition of fresh catalysts. This permitted continuous experiments for 50 h, equivalent to 70 successive batch cycles with a 94% reaction yield. However, the number of fresh catalysts required was approximated because the analyses did not enable the determination of the catalyst’s activity remaining inside the reactor. It was supposed as a mixture of precatalysts, active catalysts, and soluble deactivated catalysts with unknown ratios. The purge of deactivated catalysts could be improved by eliminating a part of the retentate before its reinjection inside the reaction. This retentate is indeed composed of these mixed catalysts and does not contain any products because they had already passed through the permeate. Therefore, it would make it possible to replace more catalysts and improve the yield stability of the reaction over time.

### 3.5. Combined Processes Using Membranes

Membrane processes have a high potential for homogeneous catalysts recovery from pharmaceutical media if the size difference between catalysts and APIs enables a steric separation. However, it could be interesting to look at coupled processes that could improve the recovery processes.

#### 3.5.1. Coupled Processes with a Liquid–Liquid Extraction

If liquid–liquid extractions result in the potential separation of catalysts from APIs, it might be interesting to couple it with a membrane process for improving the recovery. J.M. Dreimann et al. [80] developed a continuous loop for recycling rhodium catalysts for a hydroformylation reaction, combining extraction and nanofiltration. The liquid–liquid extraction process was already discussed. Thanks to the thermomorphic properties of the matrix dimethylformamide (DMF)/n-decane, most catalysts were separated in the DMF phase and directly reused. A nanofiltration step improved the process by treating the n-decane phase containing all the hydroformylation products and the rest of the catalysts. Thanks to the membrane used, most of the residual catalysts were retained while the API passed through the permeate. Thus, this optimized process enabled the continuous recovery of hydroformylation products while recirculating 97% of the initial catalyst thanks to these two successive treatments. The nanofiltration coupling permitted enabled this high recovery yield to be reached and was therefore an improvement to a simple extraction process. Even if the necessary conditions to implement such a process depend on the nature of the synthesis, couplings between thermomorphic solvents and organic nanofiltration are still studied [134].

#### 3.5.2. Membrane Adsorption

Membrane adsorption could be seen as a combined process between a membrane and an adsorbent. The literature mostly presents the application of adsorptive membranes for metal ion removal in aqueous media [135]. However, catalyst adsorption is also studied. In a review, S. Hao et al. [136] described three types of adsorbent membranes. First, homogeneous membranes are composed of a single phase (organic or inorganic) with a limited number of adsorption sites and therefore a low efficiency. Then, mixed matrix membranes—or MMMs—are composed of a polymeric matrix in which the adsorption sites are dispersed. They are easy to prepare; however, they present some disadvantages such as agglomerates of adsorbents in the polymer matrix. There can also be some sorbent loss during the desorption process which leads to contamination. J. Liu et al. [137] synthesized a hybrid chitosan/montmorillonite membrane before treating an aqueous solution containing Pd^2+^ ions. A maximum adsorbent capacity of 193 mg g^−1^ was obtained at pH 2 which is equivalent to the efficiency of a traditional adsorbent while working at ambient temperature, as described previously in this review. However, as previously discussed, the stability of polymeric materials towards organic solvents is an issue for pharmaceutical applications. The third category is composite membranes. These membranes, made of distinct layers, are more complex than the two previous ones but have the best adsorption rate [136]. Ceramic membranes with an adsorbent coating can be solvent-resistant and therefore could be used for treating a pharmaceutical medium. Membrane adsorption has advantages compared to traditional adsorbents. It does not need a filtration step and thus it is of simpler use with the possibility of implementing it in flow chemistry processes. Moreover, silica gel used as a traditional adsorbent in a stirred reactor (called adsorption in bulk) is known to cause erosion of the equipment and—when silica gel contains crystalline dust—it can represent a human hazard with risk of silicosis [138]. A fixed sorbent on a membrane coating does not have these problems. However, the desorption process stays similar to traditional adsorbents and therefore does not enable the reuse of active catalysts.

An adsorptive membrane, once loaded, can sometimes be used as a catalytic membrane if the adsorbed catalysts keep their activity [139]. The membrane is used as the reactor with a heterogeneous catalyst fixed on its surface and the separation of the synthesized molecule is permitted thanks to the filtration. However, since catalytic membranes operate as heterogeneous catalysis processes, which are not the subject of this review, this will not be further explored.

### 3.6. Comparison of Membrane Processes

**Figure 13:**
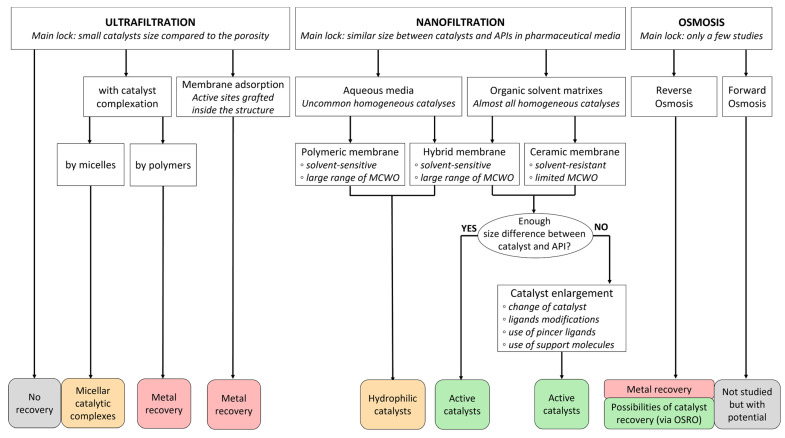
Comparative diagram for catalyst recovery by membrane processes.

Membrane processes regroup a large and complex family of technologies, some of them with great potential in the field of homogeneous catalyst recovery. Figure 13 aims to summarize the previously detailed section of this review by classifying these processes with some of their specificities and their expectations as platinoid treatments.

Ultrafiltration is mainly limited by the pore size of the membranes which is largely superior to an average catalytic complex. Thus, a catalyst complexation can be considered for enabling some recovery. The literature offers possibilities of working with functional micellar catalytic complexes which can be used in reaction cycles and isolated by membrane filtration for recycling [89,90]; however, their use is quite specific. Polymer complexation has only been conducted for metal ions as yet [91]. Another approach with UF can be performed by grafting adsorption sites on the surface or inside the membrane material during its conception for carrying out membrane adsorption processes [135,136]. As conventional adsorbents, this could be applied for treating ions or catalytic complexes but the desorption process breaks the compounds and only leads to metal recovery.

Nanofiltration membranes have a porosity more adapted towards catalyst recovery; however, in the objective of working with pharmaceutical media, the often similar size of catalysts and APIs (or intermediates) can be challenging. For the specific case of catalytic reactions in aqueous solutions, polymeric membranes can be used as they offer great flexibility in molecular-weight cutoff points. This could lead to successful catalyst recoveries; however, reactions in aqueous media in the presence of hydrophilic catalytic complexes represent a very small number of concrete cases. Most reactions are made in organic solvent matrixes which can damage polymeric membranes. Despite progress being made regarding the solvent-resistance of polymer-based materials, ceramic membranes are currently the best option for industrial purposes. However, they are limited by their MCWO. Therefore, if the size difference between catalysts and molecules of interest is enough for separation by ceramic membranes, active catalytic complexes can be recovered and reused [93,95]. If not, work could be conducted through catalyst enlargement for improving this size difference. Many approaches exist [117,126,129]—some with potential industrialization and others quite limited at the lab scale—and could lead to successful catalyst recoveries.

Osmotic processes are quite recent in the field of catalyst recovery or even metal treatment. Reverse osmosis is generally used for aqueous media and is thus only able to treat metal ions [98]. Organic solvent reverse osmosis is less common but a few results of catalyst recovery have been described in the literature [94]. Forward osmosis applied to pharmaceutical media is new. No work on catalyst recovery seems to have been made; however, this technology has been used for concentrating APIs from organic matrixes [99] and therefore has some potential.

In addition, membranes have other interesting perspectives as they can be equally implemented for batch or continuous processes [107]. It is also possible to combine them to other treatment steps such as liquid–liquid extractions for serving as a complementary treatment and improving recovery yields [80].

## 4. Conclusions

Homogeneous platinoid catalysts are almost indispensable in the pharmaceutical industry and are still central in many organic syntheses [140,141]. They offer good selectivity with mild operating conditions for powerful chemical reactions such as cross couplings which enable the formation of C–C bonding. However, their reactivity induces a sensitivity towards degradation. Thus, this kind of catalyst often degrades over time in the absence of reagents, leading to two major issues. Firstly, a need to avoid metal contamination into the active ingredients, as recommended by several standards such as the ICH Q3D(R2) [51]. Secondly, a need to recycle these catalysts based on scarce resources: in 2018, only 32% of the world palladium demand was obtained through recovery [53]. However, the regeneration of catalysts is a complex and hazardous process which must be carried out by a specialized external company, increasing the cost of the whole production chain. The use of heterogeneous catalysts is an alternative for simplifying the recovery process but it leads to a lesser reaction yield and the need for harder operating conditions, two points that could be of high importance in the pharmaceutical field. For these reasons, many industries in the fine chemistry field still prefer to avoid their use [43]. Therefore, studying homogeneous catalyst recovery and reuse is a challenging and major subject for permitting the economic viability of highly efficient syntheses while reducing the treatment processes and environmental impact of these toxic and rare catalysts.

Various conventional processes can be adapted for platinoid removal. Some of them need a pretreatment step with the breaking of the catalytic complexes into metal ions in an acidic aqueous solution. This is the case for electrochemical processes—such as electrolysis, electrodialysis, and capacitive deionization—and for some sorbents. Other technologies separate the entire catalyst through a chelation process, including most liquid–liquid extractions and adsorbents. Even though the catalyst can be removed from its medium, the reverse process is not possible and leads to the breakage of the compound and its deactivation. Only a few examples in the literature lead to a recycling process of homogeneous catalysts [80,81], often thanks to very specific conditions such as the thermomorphic properties of the reaction medium or the catalyst itself.

Membrane processes seem to be of high interest in this objective of recovering homogeneous catalysts while limiting degradation. Membranes offer the possibility to separate catalysts from pharmaceutical synthesized molecules with mild conditions while being implemented directly at the end of the synthesis—or even during a continuous process—in order to limit the degradation over time and successfully reuse it for several cycles. Some challenges are still to be overcome for extending a recovery process to all kinds of catalyzed reactions: on one side is the development of solvent-resistant materials for polymeric or hybrid membranes and on the other is the refined separation between catalysts and synthesized molecules for organic membranes through catalyst enlargement or improvement of the minimum MWCO. These approaches may lead to a successful generalization for the recycling of homogeneous catalysts.

## Figures and Tables

**Table 1:** Sales price of various catalysts on the Sigma-Aldrich (St. Louis, MI, USA) portfolio in March 2023.

bis(triphenylphosphine) palladium(II) dichloride	tris(triphenylphosphine) rhodium(I) chloride	1,1′-bis(di-isopropylphosphino) ferrocene palladium dichloride
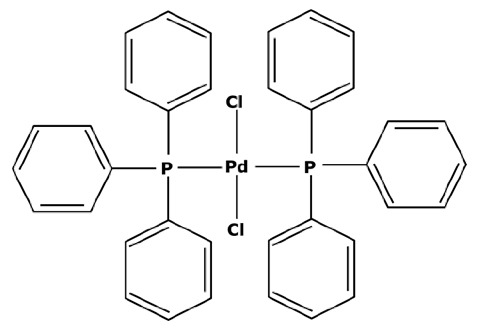	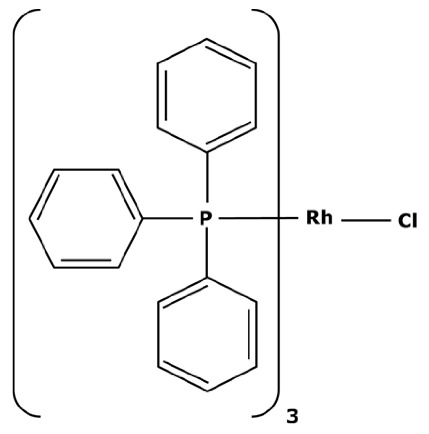	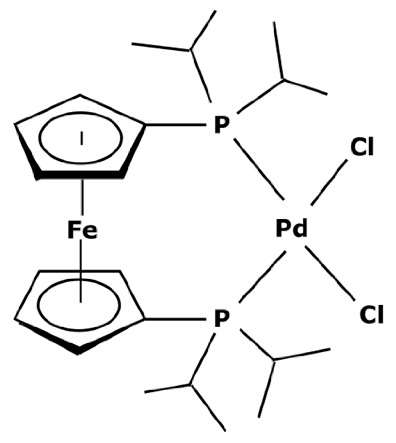
788 €/25 g	460 €/5 g	488 €/1 g

**Table 2:** Comparative table for metal treatment processes.

Processes	Specificities and Constraints		Type of Recovery
Adsorption	a preliminary dissolution into an acidic solution is often needed; possibility to recycle the sorbents after the desorption process; can be selective towards a specific metal; pH-sensitive (working at low pH).		Metal recovery
	can directly work on catalytic complexes from organic matrixes; quite expansive.		Metal recovery
Liquid–liquid extractions	a difference in solubility factors between catalysts and APIs is needed.	if induced by a chelating agent:	Metal recovery
		if there is no need of chelating agent:	Recycling of active catalytic complexes
Catalysts intrinsic properties	this only concerns very few specific catalysts.		Recycling of active catalytic complexes
Electrolysis	a preliminary dissolution into an acidic solution is needed; limited to treat ppm traces; non-selective towards a specific metal.		Metal recovery, for traces (ppm)
Electrodialysis and capacitive deionization	limited to aqueous media only and therefore metal ions; limited to treating ppm traces; current studies are limited to the lab scale.		Metal recovery, for traces (ppm)

**Table 3:** Suzuki catalysts from the Johnson Matthey portfolio with potential for a separation.

PdCl_2_(dtbpf)	PdCl_2_[P(tBu)_2_Ph]_2_	PdCl_2_(dcypf)	PdCl_2_(AmPhos)_2_	QPhosPd(crotyl)Cl
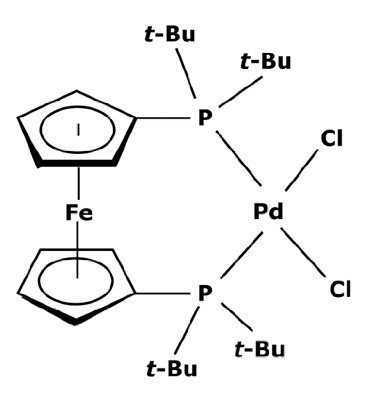	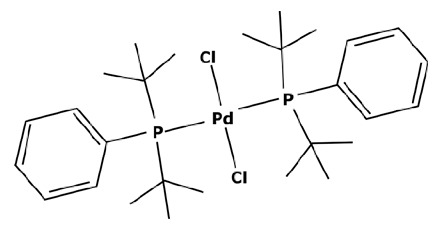	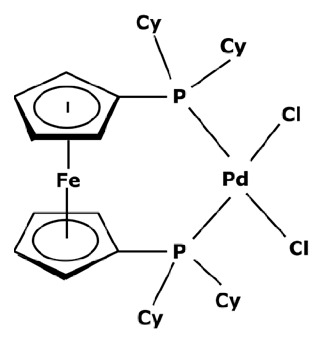	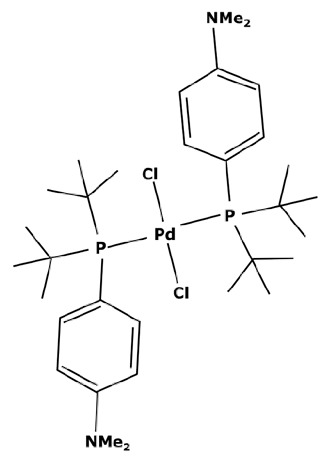	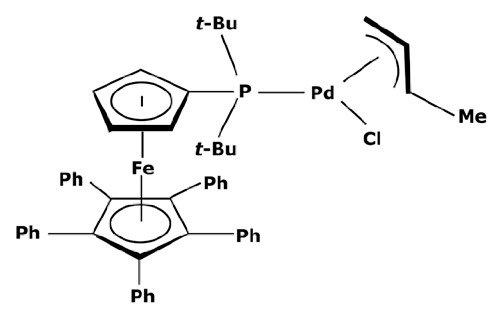
651.75 g mol^−1^	621.95 g mol^−1^	755.90 g mol^−1^	708.08 g mol^−1^	907.69 g mol^−1^
CAS: 95408-45-0	34409-44-4	917511-90-1	887919-35-9	1252598-33-6

## Data Availability

Not applicable.
